# Cloning of the RNA m^6^A Methyltransferase 3 and Its Impact on the Proliferation and Differentiation of Quail Myoblasts

**DOI:** 10.3390/vetsci10040300

**Published:** 2023-04-18

**Authors:** Jing Liu, Wentao Zhang, Wei Luo, Shuibing Liu, Hongxia Jiang, Sanfeng Liu, Jiguo Xu, Biao Chen

**Affiliations:** 1 College of Animal Science and Technology, Jiangxi Agricultural University, Nanchang 330045, China; 2 State Key Laboratory of Livestock and Poultry Breeding, Guangdong Key Laboratory of Animal Breeding and Nutrition, Institute of Animal Science, Guangdong Academy of Agricultural Sciences, Guangzhou 510000, China; 3 Institute of Biological Technology, Nanchang Normal University, Nanchang 330032, China; 4 Key Laboratory for Genetic Improvement of Indigenous Chicken Breeds of Jiangxi Province, Nanchang 330032, China

**Keywords:** quail, METTL3, myoblast, gene expression, proliferation, differentiation

## Abstract

**Simple Summary:**

RNA N6-methyladenosine (m^6^A) modification plays an important role in RNA processing, transport, translation, and stem cell differentiation. However, the function of METTL3 in quails’ skeletal muscle remains unknown. In this study, we cloned the full-length coding sequence of the quail METTL3 and examined its control of myoblast proliferation and differentiation, as well as the downstream genes and signaling pathways that it regulates. Our findings shed light on the regulatory network that governs poultry skeletal muscle growth and development.

**Abstract:**

Methyltransferase 3 (METTL3), which has been demonstrated to play a crucial role in a variety of biological processes, is the key enzyme for catalyzing m^6^A modification in RNA. However, the complete protein sequence of METTL3 in quail has not been annotated, and its function in skeletal muscle of quails remains unknown. In the current study, the full-length coding sequence of the quail *METTL3* was obtained through the 3′ rapid amplification of cDNA ends (3’ RACE) and its homology with that of other species was predicted based on a generated phylogenetic tree. A Cell Counting Kit-8 assay and flow cytometry in a quail myoblast cell line (QM7) demonstrated that METTL3 promotes myoblast proliferation. The overexpression of METTL3 in QM7 cells significantly increased the expression levels of the myoblast differentiation markers myogenin (MYOG), myogenic differentiation 1 (MYOD1), and myocyte enhancer factor 2C (MEF2C), further demonstrating that METTL3 promotes myoblast differentiation. Additionally, transcriptome sequencing following METTL3 overexpression revealed that *METTL3* controls the expression of various genes involved in RNA splicing and the regulation of gene expression, as well as pathways such as the MAPK signaling pathway. Taken together, our findings demonstrated that *METTL3* plays a vital function in quail myoblast proliferation and differentiation and that the METTL3-mediated RNA m^6^A modification represents an important epigenetic regulatory mechanism in poultry skeletal muscle development.

## 1. Introduction

Skeletal muscle growth and development are closely associated with livestock and poultry meat production, with muscle quantity being the most economically important trait in the poultry industry [1]. Quail, a type of poultry, is mainly used for egg and meat production in China (http://www.moa.gov.cn/xw/zwdt/202005/t20200529_6345528.htm, accessed on 29 May 2020). With the increase in meat consumption in this country, quail meat production performance has received increasing attention from breeders. The quail industry has broad development prospects owing to the small size, short breeding cycle, and high nutritional value of the birds [2].

N6-methyladenosine (m^6^A) modification is the most common form of RNA modification in eukaryotic cells [3]. Recent studies have shown that RNA m^6^A modification plays an important role in RNA processing, transport, and translation, as well as in stem cell differentiation [4,5,6,7,8,9]. The catalysis of m^6^A is performed by several enzymes, including methyltransferase 3 (METTL3) and METTL16 [10,11]. METTL3 recognizes specific RNA sequences and catalyzes the formation of m^6^A, thereby affecting RNA stability, metabolism, and, consequently, its post-transcriptional regulation [12]. m^6^A modification plays an important role in embryonic development, cell differentiation, and nervous system development, and has also been implicated in tumor occurrence and progression [13].

Several lines of evidence indicate that the METTL3-mediated regulation of RNA metabolism plays a role in skeletal muscle development. For instance, the loss of *Mettl3* in mice results in abnormal muscle development and impaired muscle repair [14]. In murine and bovine myoblasts, METTL3 promotes cell proliferation, while contradictory results exist regarding its role in cell differentiation [15,16,17,18]. In C2C12 cells, METTL3 was reported to enhance myogenic differentiation by mediating the m^6^A RNA modification of the key myogenic factor MYOD and by regulating the Notch signaling pathway [17,19]. Conversely, METTL3 was shown to suppress differentiation by regulating ERK signaling through the METTL3/METTL14/MNK2 axis [15]. In addition to its effects on functional genes, METTL3 has also been reported to promote the expression of muscle-specific miRNAs (miR-1a, miR-133a, miR-133b, miR-206) post-transcriptionally, thereby inhibiting the translation *n* of their target genes and suppressing muscle differentiation [16]. In primary bovine myotubes, the METTL3-m^6^A-YTHDF1 axis stabilizes MEF2C RNA and increases its translation, which promotes cell differentiation [20]. Moreover, METTL3 enhances myotube differentiation by downregulating the expression of the DNA demethylase TET1 [21]. Combined, these findings suggest that METTL3 plays a multidimensional regulatory role in myoblast differentiation; however, the underlying mechanisms remain poorly understood.

Investigating the genetic mechanisms underlying skeletal muscle development in poultry, including understanding the regulatory role of METTL3 in poultry skeletal muscle development, is vital for improving the economic benefits of the poultry industry. The aim of this study was to clone the full-length coding sequence (CDS) of quail *METTL3*, clarify its expression pattern during quail skeletal muscle development, and elucidate its regulatory role, including the potential underlying mechanisms in cell proliferation and differentiation using the QM7 cell line.

## 2. Materials and Methods

### 2.1. Animals and Cells

A total of 220 fertilized Korean Longcheng quail eggs were purchased from Hengyan Poultry Industry (Yichun, China). The eggs were incubated in a Hao Hua De incubator (Nanchang, China) at 38 °C with 60% relative humidity and were turned 12 times per day. The leg muscle tissue of quail embryos was collected daily from day 7 of incubation until hatching. Specifically, the legs were dissected from the embryos once they had been removed from the eggshell and the skin, bone, and claws were removed. The tissues were then frozen in liquid nitrogen and stored at −80 °C. The sex of each bird was determined by the PCR amplification of the chromodomain helicase DNA binding protein 1 gene (*CHD1*) using the primers listed in Table 1. The amplification was performed with Taq Master Mix (Dye Plus, Vazyme, Nanjing, China) in a T100 Thermal Cycler (Bio-Rad, Hercules, CA, USA) using the following program (20 μL reaction mixture): 95 °C for 3 min, followed by 36 cycles of 95 °C for 30 s, an annealing temperature for 30 s, and 72 °C for 60 s, with a final extension step at 72 °C for 5 min.

The QM7 cell line used in this study was obtained from our college. The cells were cultured in M199 medium (Cytiva, MA, USA) containing 10% fetal bovine serum (Gibco, Grand Island, NE, USA), 10% tryptose phosphate broth solution (Sigma, St. Louis, MO, USA), and 0.5% penicillin/streptomycin (Gibco) at 37 °C with 5% CO_2_ in a humidified cell culture incubator (BB150; Thermo Scientific, Waltham, MA, USA). At 90%~100% confluence, the cells were detached in 1 mL of 0.25% trypsin solution for 1 min, followed by the addition of 4 mL of complete medium to halt digestion. The cells were then dissociated into single-cell suspensions by gentle tapping and collected by centrifugation at 1000 rpm for 5 min for sub-culture or plating. After achieving 90% confluency, the QM7 were induced to differentiation with M199 medium supplemented with 2% horse serum. Following the induction of differentiation, QM7 cells were collected daily up to the fifth day (DM1–DM5).

### 2.2. RNA Extraction and 3′ Rapid Amplification of cDNA Ends (RACE) PCR

RNA was isolated from tissues and cells using the FastPure Cell/Tissue Total RNA Isolation Kit (Vazyme). First-strand cDNA for 3′ RACE was synthesized using the SMART RACE cDNA Amplification Kit (Clontech, Otsu, Japan) with the RNA from quail leg muscle as a template. The gene-specific primers were designed based on the NCBI reference sequence XM_015850477.2 using Oligo 7 and were synthesized by TsingKe (Changsha, China) (Table 1). RACE-PCR was performed using the KOD FX (Toyobo, Osaka, Japan) touch-down PCR program as follows: initial denaturation at 94 °C for 2 min; 5 cycles of 98 °C for 10 s and 74 °C for 2 min; 5 cycles of 98 °C for 10 s and 72 °C for 2 min; 5 cycles of 98 °C for 10 s and 70 °C for 2 min; and 25 cycles of 98 °C for 10 s and 68 °C for 2 min, followed by a final extension at 68 °C for 7 min and storage at 4 °C. The PCR products were purified using the FastPure Gel DNA Extraction Mini Kit (Vazyme), according to the manufacturer’s instructions, and were then cloned into the pJET 1.2/Blunt vector (Thermo Scientific) for Sanger sequencing (Tsingke). The sequencing results were assembled and analyzed using DNASTAR SeqMan (https://www.dnastar.com/, accessed on 2 July 2021) and the phylogenetic tree was constructed using DNAstar MegAlign.

### 2.3. cDNA Synthesis and Fluorescence Quantitative PCR (qPCR)

Total RNA extracted from cells or tissues was reverse transcribed using the HiScript II 1st Strand cDNA Synthesis Kit with gDNA wiper (Vazyme), according to the manufacturer’s instructions. The synthesized cDNA was diluted at a ratio of 1:4 and used for subsequent qPCR analysis. qPCR was performed using 2× T5 Fast qPCR Mix (Tsingke) on an ABI Q5 Real-Time PCR System (Thermo Scientific). The qPCR steps were as follows (10 µL reaction volume): 95 °C for 3 min, followed by 40 cycles of 95 °C for 10 s and annealing temperature for 1 min, and melt-curve analysis was performed at 65–95 °C. Three technical replicates were performed for each sample, and the quantification results were calculated using the 2^−∆∆Ct^ with quail *GAPDH* serving as the internal control. The results are presented as means ± S.E.M. The primers were designed using NCBI Primer-BLAST (https://www.ncbi.nlm.nih.gov/tools/primer-blast/, accessed on 10 January 2022) and the sequences are shown in Table 1.

### 2.4. Vector Construction and Small Interfering RNA (siRNA) Synthesis

The assembled *METTL3* CDS was synthesized by Tsingke and inserted into the pEGFP-C1-3 × flag vector at the BglII and HindIII restriction sites. The Endo-Free Plasmid DNA Mini Kit II (Omega, Guangzhou, China) was used for plasmid purification (METTL3 and negative control). siRNAs targeting quail *METTL3* were designed using BLOCK-iT RNAi Designer (https://rnaidesigner.thermofisher.com/rnaiexpress/sort.do, accessed on 16 January 2022) and synthesized by Tsingke. The target sequences are shown in Table 1. Cell transfection was performed with Lipo8000 according to the manufacturer’s instructions (Beyotime, Shanghai, China). The amount of transfection reagent used was 5/8 of the recommended amount.

### 2.5. Western Blotting

Western blotting was performed as previously described [22], with minor modifications. Cells were lysed in radioimmunoprecipitation assay (RIPA) buffer containing phenylmethanesulfonyl fluoride (PMSF) (Beyotime) for 5 min, centrifuged at 12,000 rpm for 10 min at 4 °C, and the supernatant was then collected. Protein was separated by SDS–PAGE, blotted onto nitrocellulose membranes (Merck Millipore, Billerica, MA, USA) and labeled with primary and secondary antibodies. The primary antibody was anti-DYKDDDDK (1:10,000; M20008, Abmart, Shanghai, China) and the secondary antibody was HRP-conjugated goat anti-mouse lgG (1:20,000; Signalway Antibody, MD, USA). The gel was exposed, and images were captured on an Amersham Imager 600 (GE Healthcare, Uppsala, Sweden).

### 2.6. Cell Cycle Analysis

QM7 cells were seeded in 12-well plates and transfected with pEGFP-C1-3 × flag-METTL3, pEGFP-C1-3 × flag, si-METTL3, or si-NC (negative control) using Lipo8000. After 6 h of transfection, the cells were refreshed with complete medium and harvested after 42 h. Cell cycle stage analysis was performed by flow cytometry as previously described [23].

### 2.7. Cell Counting Kit-8 (CCK-8) Assay

QM7 cells were seeded in 96-well plates and transfected with pEGFP-C1-METTL3, pEGFP-C1, si-METTL3, or si-NC, and cell proliferation was evaluated every 12 h using CCK-8 reagent (Meilunbio, Dalian, China). Specifically, 10 μL of CCK-8 solution was added to each well, and the cells were incubated for 2 h at 37 °C in a CO_2_ cell culture incubator. The absorbance of each well at 450 nm was measured using a microplate reader (Infinite 200 PRO, Tecan, Männedorf, Switzerland). After the assay, the cells were washed two or three times with PBS and then replenished with complete medium.

### 2.8. RNA-seq and Data Analysis

QM7 cells were seeded in 12-well plates, transfected with pEGFP-C1-METTL3 or pEGFP-C1 for 6 h, and then cultured in fresh complete medium for 42 h. RNA was subsequently extracted from the cells using the FastPure Cell/Tissue Total RNA Isolation Kit. Total RNA quantity and quality were determined using a Nanodrop ND-1000 spectrophotometer (NanoDrop, Wilmington, DE, USA) and a Bioanalyzer 2100 (Agilent, CA, USA), respectively. Poly(A) RNA sequencing libraries were generated and paired-end (PE150) sequenced using an Illumina Novaseq 6000 System (LC Bio, Hangzhou, China) according to standard protocols. Clean reads were mapped to the *Coturnix japonica* reference genome (https://www.ncbi.nlm.nih.gov/genome/113?genome_assembly_id=265191, accessed on 20 June 2022) and subjected to subsequent analysis following previously described methods [24]. Gene set enrichment analysis (GSEA) was conducted as previously described [25]. Pathways with |normalized enriched scores (NESs)| > 1, NES *p*-values < 0.05, and false discovery rate (FDR) *q*-values < 0.05 were considered significantly enriched.

## 3. Results

### 3.1. Quail Sex Determination

**Figure 1:**
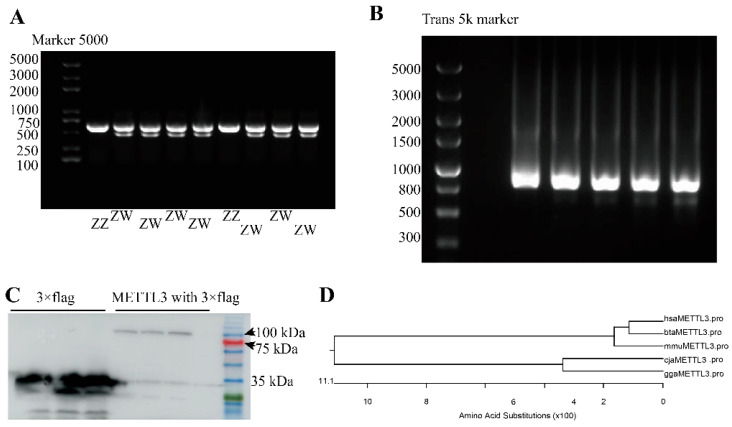
Cloning and sequence analysis of the coding sequence (CDS) of quail *METTL3*. (**A**) PCR products of *CHD1* gene amplification. The products observed in different lanes are derived from distinct quail individuals. (**B**) Agarose gel electrophoresis of 3′ RACE-PCR products. The products observed in different lanes represent the same product obtained from various PCR tubes. (**C**) The western blotting analysis of the METTL3-3 × flag fusion protein and the negative control. The initial three lanes depict the protein products of blank vector with 3 × flag and eGFP-fused, whereas the latter three lanes represent the protein products of quail METTL3 fused with 3 × Flag and eGFP. (**D**) The phylogenetic tree generated for METTL3. (Please find the WB full membrane and the full images of agarose gel electrophoresis of Figure 1 in Figure S1).

The quail *CHD1* gene is located on the sex chromosome. Here, the agarose gel electrophoresis of *CHD1* amplicons identified 63 female quail embryos (two bands (638 and 470 bp) and 55 male quail embryos (one band (638 bp)) (Figure 1A, Table 2).

### 3.2. Cloning and Sequence Analysis of the Full-Length CDS of METTL3

Before constructing the expression vector, we compared the size of the METTL3 protein of quail in the NCBI database (XP_015705963.1, 477 aa) with that of chicken (XP_040510970.1, 574 aa), human (NP_062826.2, 580 aa), mouse (NP_062695.2, 580 aa), and cattle (NP_001095708.1, 580 aa) and found it to be smaller. This suggested that the CDS of quail *METTL3* was incomplete; therefore, we subsequently amplified the full length of *METTL3* using RACE-PCR and obtained an 889-bp sequence (Figure 1B). After removing the part that overlapped with the known sequence, we obtained a new 3′ sequence of 384 bp for quail *METTL3* (Supplementary File S1). The splicing of the sequence obtained by 3′ RACE yielded the new RNA for *METTL3*, which contained a CDS region of 1683 bp that was predicted to encode a protein of 560 aa (Supplementary File S1). Based on the results of Western blotting analysis, it was determined that the size of the quail METTL3 protein after fusion with 3 × flag and eGFP was approximately 100 kDa. In comparison, the size of the 3 × flag protein fused with eGFP in the empty vector was approximately 30 kDa. Thus, we can infer that the size of the quail METTL3 protein is approximately 70 kDa (Figure 1C). The predicted protein was closer in length and size to the METTL3 proteins of chicken, human, mouse, and cattle, while the protein sequence of quail METTL3 was closer to that of chicken than to that of humans, mice, and cattle METTL3, which was expected (Figure 1D). These observations demonstrated that the obtained CDS of quail *METTL3* was the full-length CDS.

### 3.3. Expression Patterns of Differentiation Markers and METTL3 during Quail Embryo Leg Muscle Development

**Figure 2:**
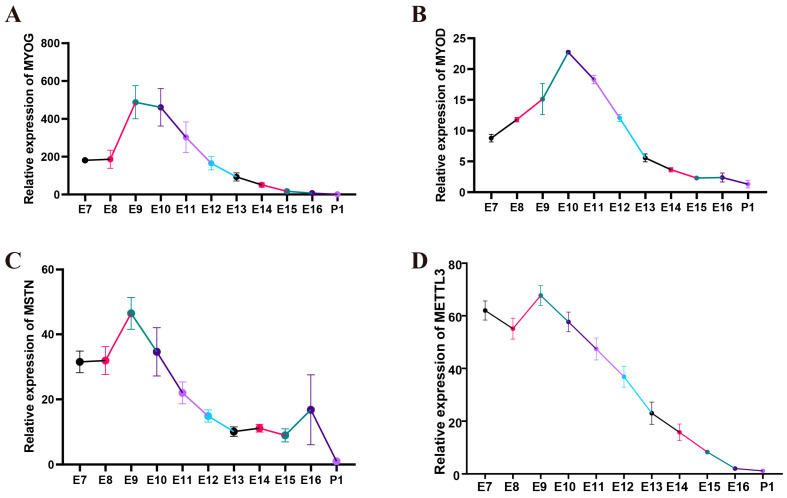
The spatiotemporal expression of skeletal muscle differentiation marker genes and *METTL3* in quail leg muscle. (**A**–**D**) Spatiotemporal expression of *MYOG*, *MYOD*, *MSTN*, and *METTL3* from E7 to P1 in leg muscle. E7–E16 represents embryonic day 7 to embryonic day 16, and P1 represents post-hatch day 1. Four female quails were used at each time point. Different colors in the figure indicate the different developmental stages of quail leg muscle.

To investigate the key events of quail myoblast differentiation, we extracted total RNA from leg muscles at various stages of embryonic development and used qPCR to determine the expression patterns of the differentiation marker genes *MYOG* and *MYOD* and the muscle-inhibiting factor *MSTN*. In order to identify patterns of gene expression, four female quail individuals were used at each time point. The results showed that *MYOG* expression remained low at E7 and E8, significantly increased from E8 to E9, remained at a high level from E9 to E10, and then gradually decreased from E10 (Figure 2A). *MYOD* expression gradually increased from E7 to E10 and significantly decreased from E10 (Figure 2B). *MSTN* expression remained low at E7 and E8, significantly increased from E8 to E9, and, subsequently, gradually decreased from E9 (Figure 2C). The expression level of *METTL3* during quail leg muscle development was relatively low at E7 and E8, peaked at E9, and gradually decreased during subsequent embryonic development (Figure 2D). These results suggested that *METTL3* expression gradually decreases during quail leg muscle differentiation.

### 3.4. The Effects of METTL3 on QM7 Cell Proliferation

**Figure 3:**
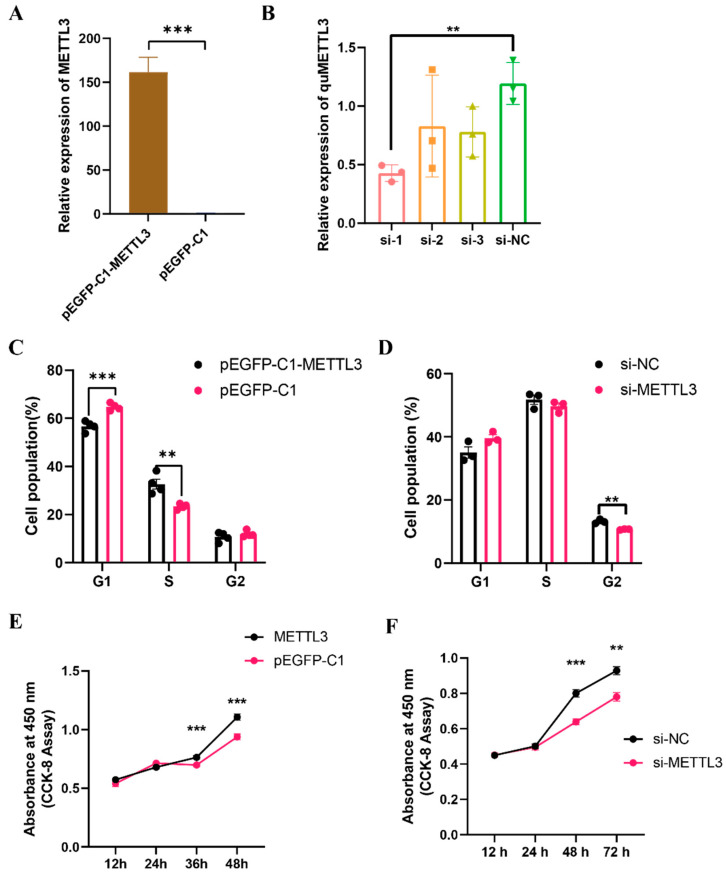
*METTL3* enhances the proliferative ability of QM7 cells. (**A**,**B**) The relative expression level of *METTL3* after the overexpression (**A**) and knockdown (**B**) of METTL3. (**C**) Cell cycle analysis of QM7 cells after the transfection of pEGFP-C1-METTL3 or pEGFP-C1. (**D**) Cell cycle analysis of QM7 cells after the transfection of siRNA targeting *METTL3* (si-1) or the negative control (si-NC). (**E**,**F**) CCK-8 assay in QM7 cells after the overexpression or knockdown of *METTL3*. ** indicates *p* < 0.01; *** indicates *p* < 0.001.

To investigate the role of *METTL3* in QM7 cell proliferation, we first validated the efficiency of *METTL3* overexpression and knockdown in QM7 cells. Quantitative analysis showed that the expression of the *METTL3* gene in cells transfected with the overexpression vector (pEGFP-C1-METTL3) was significantly higher (an approximately 150-fold increase, *p* < 0.001) than that in the control group (Figure 3A). Of the three siRNAs we designed, si-1 achieved an efficiency of over 50% in knocking down *METTL3* gene expression (Figure 3B) and was thus used in subsequent *METTL3* knockdown experiments. The flow cytometric analysis of the cell cycle in QM7 cells overexpressing *METTL3* showed that, compared with the negative controls, there was a significant decrease (*p* < 0.001) in the proportion of cells in the G1 phase and a significant increase (*p* < 0.01) in that of cells in the S phase compared with that in the negative control group (Figure 3C). Conversely, the knockdown of *METTL3* resulted in a significant decrease (*p* < 0.01) in the proportion of cells in the G2 phase (Figure 3D), indicating that *METTL3* can accelerate cell cycle progression. CCK-8 assay results showed that *METTL3* overexpression significantly increased (*p* < 0.001) the proliferative ability of QM7 cells (Figure 3E), while the knockdown of *METTL3* exerted the opposite effect (*p* < 0.01) (Figure 3F). These results indicate that the *METTL3* gene enhances cell proliferation in QM7 cells.

### 3.5. The Regulatory Effect of METTL3 Protein in QM7 Cell Differentiation

**Figure 4:**
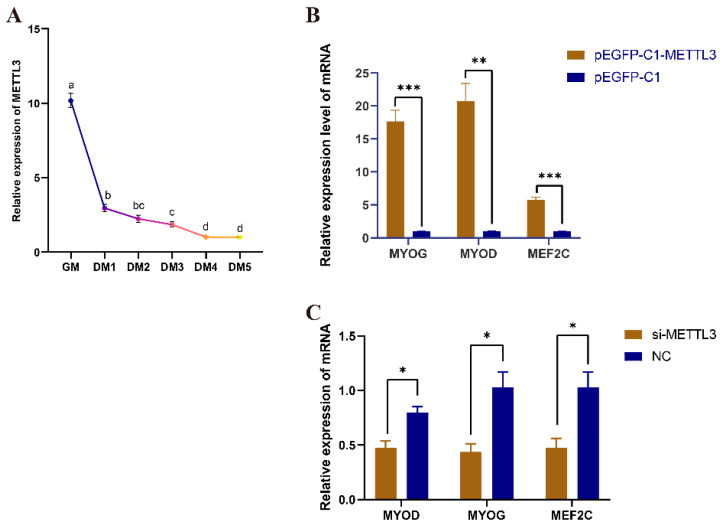
METTL3 promotes the differentiation of QM7 cells. (**A**) The expression level of *METTL3* before and after QM7 cell differentiation. Different colors indicate the different differentiation stages of QM7. The significant differences between the two groups are indicated by the distinct letters (*p* < 0.05). (**B**) The expression levels of *MYOG*, *MYOD*, and *MEF2C* after the overexpression of *METTL3*. (**C**) The expression levels of *MYOG*, *MYOD*, and *MEF2C* after the knockdown of *METTL3*. GM represents growth medium and the cells undergo proliferation, whereas the differentiation medium (DM) phases, DM1–DM5, represent the first to fifth day of differentiation. * indicates *p* < 0.05; ** indicates *p* < 0.01; *** indicates *p* < 0.001.

The mRNA expression level of METTL3 gradually decreased during the proliferation phase (GM) and various stages of differentiation (DM1–DM5) in QM7 cells (Figure 4A), suggesting that METTL3 plays an important role in myoblast differentiation. The overexpression of *METTL3* significantly increased the expression levels of myogenic and fusion marker genes (*MYOG*, *MYOD*, and *MEF2C*) (Figure 4B), whereas the opposite was seen with *METTL3* knockdown (Figure 4C). These results indicated that *METTL3* can promote differentiation in QM7 cells.

### 3.6. The Effects of METTL3 Overexpression on the Transcriptome of QM7 Cells

**Figure 5:**
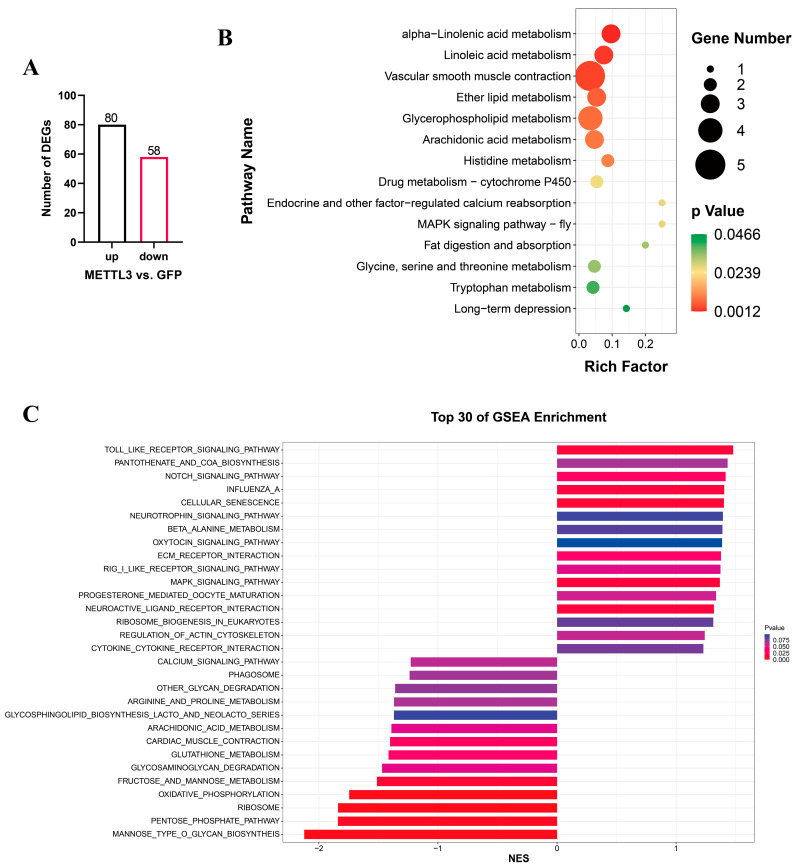
RNA-seq analysis in QM7 cells overexpressing *METTL3*. (**A**) The number of differentially expressed genes (DEGs) after *METTL3* overexpression. (**B**) KEGG pathway enrichment analysis relating to the differentially expressed genes. (**C**) The top 30 enriched KEGG pathways based on GSEA with the RNA-seq data.

After determining the effect of METTL3 overexpression in QM7 cells, cells in the overexpression and negative control groups were subjected to poly(A)-tail library sequencing. Differential gene expression analysis identified a total of 138 differentially expressed genes, 80 of which were upregulated and 58 downregulated (Figure 5A, Table S1). KEGG pathway enrichment analysis showed that the differentially expressed genes were significantly enriched in 14 signaling pathways, including those related to lipid metabolism, vascular smooth muscle contraction, and MAPK signaling pathway-fly (Figure 5B, Table S2). LOC107314843, LOC107320189, LOC107314846, receptor activity modifying protein 2 (RAMP2), and *RAMP3* were enriched on vascular smooth muscle contraction. Docking protein 5 (DOK5) was enriched on MAPK signaling pathway-fly. It is noteworthy that the gene exhibiting the highest expression level and most significant differential expression among all genes was the Heat shock protein beta-9 (HSPB9). In addition, GSEA was used to analyze the overall effect of *METTL3* on the transcriptome of QM7 cells. The results indicated that significant changes occurred in the MANNOSE_TYPE_O_GLYCAN_BIOSYNTHEIS, RIBOSOME, and PENTOSE_PHOSPHATE_PATHWAY signaling pathways (|NES| > 1, NOM.pval < 0.05, FDR.qval < 0.05, Figure 5C, Table S3). In the RIBOSOME signaling pathway, the expression of RPL and RPS family genes related to ribosome function, RNA processing, and translation was significantly altered (Table S4).

## 4. Discussion

The m^6^A modification mediated by METTL3 is known to play multiple roles in muscle development [26]; however, whether it exerts a regulatory function in avian myoblast proliferation and differentiation is unknown. In this study, we performed 3′ RACE using *METTL3* gene-specific primers designed based on the predicted sequence of quail *METTL3* mRNA in the NCBI database (XM_015850477.2). The 3′ RACE product was cloned, sequenced, and spliced, yielding the predicted mRNA sequence of *METTL3*. However, despite multiple primer design attempts, we were unable to obtain results for 5′ RACE. Sequence analysis showed that the predicted 1823 bp METTL3 mRNA containing a CDS of 1683 bp that was predicted to encode a 560 aa amino acid protein similar in length to METTL3 proteins from humans, mice, cows, and chickens. Western blotting analysis indicated that the protein had a molecular mass of approximately 100 kDa with the 3× flag-eGFP and approximately 70 kDa without the 3× flag-eGFP, which was similar to that seen for METTL3 proteins from other species. Homology analysis results indicated that the obtained CDS represented the full-length CDS of quail *METTL3* and was, therefore, used in subsequent cellular experiments.

Upon proliferation, migration, and contact with other cells, myogenic cells undergo a cessation of proliferation and transition into a state of cell cycle arrest. They then express the myogenic regulatory factors MYOG and MYOD to promote differentiation into myocytes, which migrate, recognize each other, and ultimately fuse into myotubes [27]. MSTN is a muscle growth inhibitor the expression of which initially increases, and then decreases, during avian muscle differentiation [28,29]. To better understand the changes in *METTL3* expression during quail leg muscle development, we first explored the spatiotemporal expression patterns of differentiation marker genes in quail leg muscle to identify developmental turning points. *MYOG*, *MYOD*, and *MSTN* all showed a trend of an initial increase in expression, followed by a decrease, with expression peaking at E9 and E10. This suggested that quail leg muscle differentiation begins at E8, peaks at E10, and is mostly completed by E13. The expression of *METTL3* in undifferentiated quail leg muscle was relatively high and gradually decreased starting at E9, indicating that *METTL3* expression decreases with increasing muscle differentiation. This result was consistent with the spatiotemporal expression patterns of *METTL3* in the hindlimbs of mice, suggesting that a decrease in *METTL3* expression is necessary for quail skeletal muscle differentiation [15].

Studies have demonstrated that METTL3 regulates myoblast proliferation and differentiation by modulating multiple genes and pathways through m^6^A RNA modification [18,21]. METTL3 enhances cell proliferation by elevating the m^6^A modification level of p21 and p27 mRNA, which shortens their half-lives [18]. METTL3 also promotes myoblast differentiation by increasing the stability of the mRNA of myogenic regulatory factors such as MyHC, MYOD, and MYOG through m^6^A modification [18]. In addition, METTL3 was shown to promote the differentiation of C2C12 cells by regulating the Notch signaling pathway [17]. Furthermore, METTL3 can suppress the expression of the DNA demethylase TET1 through m^6^A modification, which, in turn, promotes METTL3 expression by mediating the demethylation of the *METTL3* DNA region, leading to the regulation of the m^6^A modification of the entire genome and transcriptome and the promotion of myoblast differentiation [21]. In bovine myoblasts, the addition of the methylation inhibitor 3-Deazaadenosine (DAA) reduces the expression of MEF2C protein, and interference with METTL3 also inhibits the expression of MEF2C protein, indicating that RNA m^6^A modification helps to stabilize *MEF2C* mRNA, promote its protein expression, and, thus, promote myogenic differentiation [20]. In contrast, in C2C12 cells, METTL3 can inhibit myoblast differentiation by increasing the expression of muscle-specific miRNAs [16]. In complex with METTL14, METTL3 also mediates the m^6^A modification of MNK2, thereby exerting regulatory effects on the ERK signaling pathway. The knockout of both METTL3 and METTL14 promotes myoblast differentiation [15]. We speculate that METTL3 regulates myoblast differentiation through multiple dimensions, which may be due to the overexpression and interference efficiency during experimentation in different papers. The effects of genes on the same process may vary depending on the species, culture conditions, and regulatory dimensions. Different experiments adopt varying treatments, transfection reagents, and gene expression methods, which can result in inconsistent experimental results. The most reliable outcomes can be obtained by knocking out a specific gene in vivo level. METTL3’s regulation of m^6^A modification is characterized by changes in the overall transcriptome modification level, with knockdown being less effective than overexpression. Here, we found that, in QM7 cells, METTL3 accelerates the cell cycle, promotes proliferation, and enhances differentiation.

To further investigate the potential mechanisms underlying the METTL3-mediated regulation of QM7 cell proliferation, we overexpressed *METTL3* and then performed an RNA-seq analysis, with pEGFP-C1-transfected cells serving as a negative control. RNA-seq data analysis revealed significant changes in the expression of 138 genes in *METTL3*-overexpressing cells. KEGG pathway enrichment analysis showed that the differentially expressed genes were significantly enriched in the MAPK signaling pathway-fly. GSEA similarly showed significant changes in the expression of genes in the MAPK signaling pathway. Cellular processes, such as cell differentiation, cell division, cell proliferation, metabolism, and apoptosis, are regulated by the MAPK signaling pathway [30]. MAPK signaling plays a crucial role in myogenesis [31]. These observations are consistent with a previous RNA-seq analysis on C2C12 cells overexpressing METTL3 in which a significant enrichment of differentially expressed genes in the MAPK signaling pathway was reported [15]. Interestingly, our RNA-seq results, as well as previous studies in C2C12 cells overexpressing METTL3, showed the enrichment of partially overlapping signaling pathways such as the notch signaling pathway and ECM receptor interaction, suggesting that METTL3 regulates multiple genes and signaling pathways. The RIBOSOME pathway, which plays an important role in cell cycle regulation, RNA binding, and mRNA translation [32], was also significantly enriched in METTL3-overexpressing QM7 cells in our study. The regulation of the RIBOSOME pathway by METTL3 may be one dimension of its multidimensional regulatory function. Notably, our RNA-seq analysis of QM7 cells overexpressing METTL3 showed primarily the enrichment of regulatory genes and pathways associated with cell proliferation. The mechanisms underlying the METTL3-mediated regulation of QM7 cell differentiation require further exploration.

## 5. Conclusions

Gene cloning and sequence analysis showed that the quail METTL3 protein has high homology with its mammalian and chicken counterparts. Additionally, quail METTL3 was found to promote the proliferation and differentiation of skeletal muscle cells.

## Figures and Tables

**Table 1:** The information of primers and small interfering RNAs.

Name	Sequence (5′–3′)	Tm (°C)	Product Size	Usage
quCHD1-F	CCATACCTCTGATCCTTCTGC	57	470 or 638	Sexual determination
quCHD1-R	CAAGTTACTGATTCGTCTGCG	57		
quMETTL3-3-G1	AGCCGACCGGCCCTGCAGGAAGTTGCATTTCCGTCGCATC	/	/	Gene specific primer of the 3′RACE of quail METTL3
quMYOG-q-F	CTCCAAGCTGGAAATGGGGT	60	109	qPCR primers of quail MYOG
quMYOG-q-R	GGATTTGGGCCGTTTCAGTG	60		
quMYOD1-q-F	AACTGCTCCGATGGCATGAT	60	149	qPCR primers of quail MYOD1
quMYOD1-q-R	CTTGAAAGGCAGTCGAGGCT	60		
quaMSTN-q-F	CGTGAGATCCACCACTTCGT	60	112	qPCR primers of quail MSTN
quaMSTN-q-R	AGGATGTTGGCAATGCCTAGT	60		
quMETTL3-q-F	TACGGCACCTTGACCGACGA	59.7	95	qPCR primers of quail METTL3
quMETTL3-q-R	ATGGCTCTTCCCGTGACC	59.9		
quMEF2C-q-F	CCACTGGCCCATCCTTCTTT	60	154	qPCR primers of quail MEF2C
quMEF2C-q-R	AGTTGCGGGGATTGCCATAA	60		
METTL3 si-1	GAGCTCCATTCAGGCCCATAAGAAA	/	/	Small interfering RNA 1 of quail METTL3
METTL3 si-2	CGTGGATCTGGAGATTGAGAGTGTA	/	/	Small interfering RNA 2 of quail METTL3
METTL3 si-3	ACCTGGACGTGAGCATTCTGGGTAA	/	/	Small interfering RNA 3 of quail METTL3

**Table 2:** Count of quail embryos by sex.

	E7	E8	E9	E10	E11	E12	E13	E14	E15	E16	P1	Total
ZZ (male)	2	3	4	9	7	6	4	5	3	3	9	55
ZW (female)	7	4	10	3	4	4	6	5	7	7	6	63

## Data Availability

All the RNA sequencing data and processing files can be found on the China National Center for Bioinformation website, under accession number PRJCA015359. The information will be released on 4 March 2025.

## Supplementary Materials

The following supporting information can be downloaded at: https://www.mdpi.com/article/10.3390/vetsci10040300/s1, Supplementary File S1: the sequence of 3′RACE product and the splicing mRNA of quail METTL3; Table S1: the list of the differentially expressed genes in METTL3 overexpression group vs. negative control group; Table S2: the KEGG analysis with all differentially expressed genes in METTL3 overexpression group vs. negative control group; Table S3: the GSEA KEGG analysis of the gene expression profile in METTL3 overexpression group vs. negative control group; Table S4: the GSEA details of RIBOSOME pathway; Figure S1: the WB full membrane and the full images of agarose gel electrophoresis of Figure 1.
