# Supplementary material for: Cloning of the RNA m6A Methyltransferase 3 and Its Impact on the Proliferation and Differentiation of Quail Myoblasts
Source: Vet Sci. 2023 Apr 18;10(4):300. doi: 10.3390/vetsci10040300 (PMC10144998; doi:10.3390/vetsci10040300)
Supplement: Supplementary file 1 [file vetsci-10-00300-s001.zip › Supplementary file S1.pdf]

Quail METTL3 3'RACE

tggcgttaagggcaaccctcaaggcttcaaccgcggcctggattgcgacgtcatcgtagccgaggttcgttccaccagcca
taaacccgatgaaatctatgggatgatcgaacgcctttcgcccggaacccgtaaaatcgaactcttcggtcgaccccataac
gtccaaccgaattggatcactttaggaaaccaattggacgggattcatctcttggaccccgacgtcgtggcgcaatttaaaca
acattacccggatggaatcatctcaaaacccaaaaacatgtaaattttaacacccgttttggggccgtttacccaaaaatgatc
atttttacccctgatgatccaaaaaacacaaaaaaaaaaaaaaaaaaaaaaaagt

Quail METTL3 full mRNA (with CDS italic)

gggtcggaagcgggaaggggctctgaggtggaaggcggggccgcc*atgtctgatacctggagctccattcaggccca*
taagaaacaattggattcgctgagggagcggctgcagaggaggaggaagcaggacccgctcgacccccgtcacac
cgacccctctcaaccccccccctcccctccccacccaccctcctccattccacccttccagctctgtaggaagttccatctc
aacccctgggggggttgtgggggtctctaccaccccatctacggatgaagggggtccggaattgaccccagacccag
ctttagaacgtcgattgctgcttcacctcgccgatgtcgccctcactttgcccaccgatgccggagcgatacggggggct
atcgatacgcccgaaacgccggcgacgcacaacatggtggaaagcctcctccagaagttcgcggcccaggagctga
tcgaagtacgtcgcggcctattggccgaaggccccaccatcgtcacctacgccgaccactccaagttggcggccatga
caggctccgaacgaggccgcgacatggaggccgaagtcggcgggaggaagaggagagcggaacaggaaatgtg
cggcggggtaggaggaaacggacaggaagtggggaaggaagtggtgaagaaatggcggaaacaagcgtccgac
gtggatctggagattgagagtgtaagccaggagatcctggagctgctgaacaccaccacggccaaggagcaatccat
cgtggagaagttccgctcgcggggtcgggcccaggtccaggaattctgtgaccacggaaccaaagaggaatgcatc
aaagccaccggagccgaccggccctgcaggaagttgcatttccgtcgcatcatcaacaaacacacggacgaatcttt
gggcgactgctccttcctcaacacctgcttccacatggacacctgtaaatacgtccactacgagatcgacgcctgttccg
agacccccgccgagaccaccgccccaccgggtcgagaacacggaccggaactacccaggtcacaacccgacgcc
ggcgccgaccgactcttcccaccccagtggatctgctgcgacatccgctacctggacgtcagcatcctgggtaagtttgc
ggtggtgatggccgaccccccgtgggacattcacatggagctgccctacggcaccttgactgacgacgagatgaggc
ggctcaacatccccgtcctacaagacgagggattcctcttcctatgggtcacgggaagagccatggagttgggccggg
aatgcctgaacctatgggggtacgagcgtgtggacgagatcatctgggtgaagaccaaccagctgcagcgcatcatc
cgcacgggacggacgggacattggctgaaccacggcaaggagcattgtctggttggcgttaagggcaaccctcaag
gcttcaaccgcggcctggattgcgacgtcatcgtagccgaggttcgttccaccagccataaacccgatgaaatctatgg
gatgatcgaacgcctttcgcccggaacccgtaaaatcgaactcttcggtcgaccccataacgtccaaccgaattggatc
actttaggaaaccaattggacgggattcatctcttggaccccgacgtcgtggcgcaatttaaacaacattacccggatg
*gaatcatctcaaaacccaaaaacatgtaa*attttaacacccgttttggggccgtttacccaaaaatgatcatttttacccctg
atgatccaaaaaacacaaaaaaaaaaaaaaaaaaaaaaaagt

Quail METTL3 protein sequence (560 aa)

MSDTWSSIQAHKKQLDSLRERLQRRRKQDPLDPRHTDPSQPPPPLPTHPPPFH
PSSSVGSSISTPGGVVGVSTTPSTDEGGPELTPDPALERRLLLHLADVALTLPTD
AGAIRGAIDTPETPATHNMVESLLQKFAAQELIEVRRGLLAEGPTIVTYADH
SKLAAMTGSERGRDMEAEVGGRKRRAEQEMCGGVGGNGQEVGKEVVKKW
RKQASDVDLEIESVSQEILELLNTTTAKEQSIVEKFRSRGRAQVQEFCDHGTKE
ECIKATGADRPCRKLHFRRIINKHTDESLGDCSFLNTCFHMDTCKYVHYEIDA
CSETPAETTAPPGREHGPELPRSQPDAGADRLFPPQWICCDIRYLDVSILGKFAV
VMADPPWDIHMELPYGTLTDDEMRRLNIPVLQDEGFLFLWVTGRAMELGRE
CLNLWGYERVDEIIWVKTNQLQRIIRTGRTGHWLNHGKEHCLVGVKGNPQGF
NRGLDCDVIVAEVRSTSHKPDEIYGMIERLSPGTRKIELFGRPHNVQPNWITLG
NQLDGIHLLDPDVVAQFKQHYPDGIISKPKNM.
