# Supplementary figures and images for: Cloning of the RNA m6A Methyltransferase 3 and Its Impact on the Proliferation and Differentiation of Quail Myoblasts
Source: Vet Sci. 2023 Apr 18;10(4):300. doi: 10.3390/vetsci10040300 (PMC10144998; doi:10.3390/vetsci10040300)

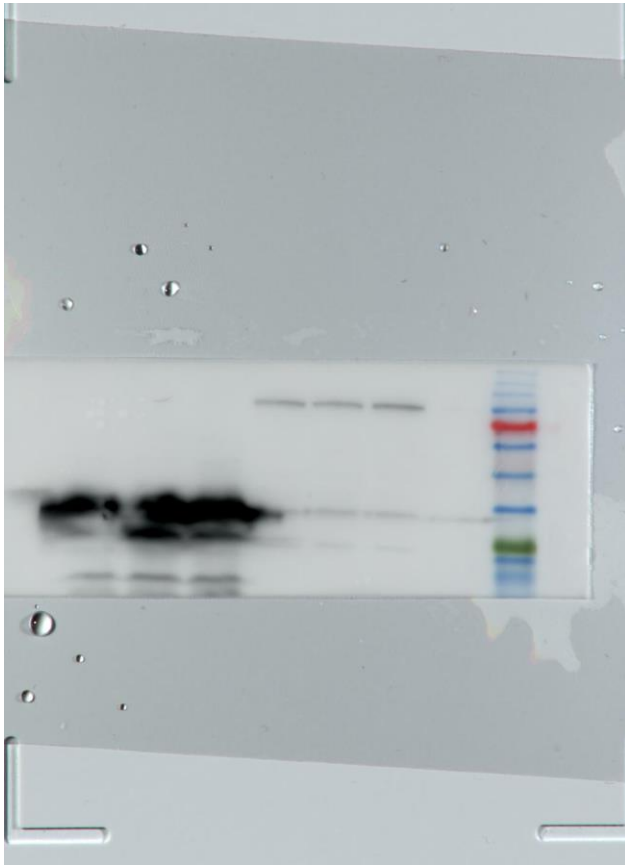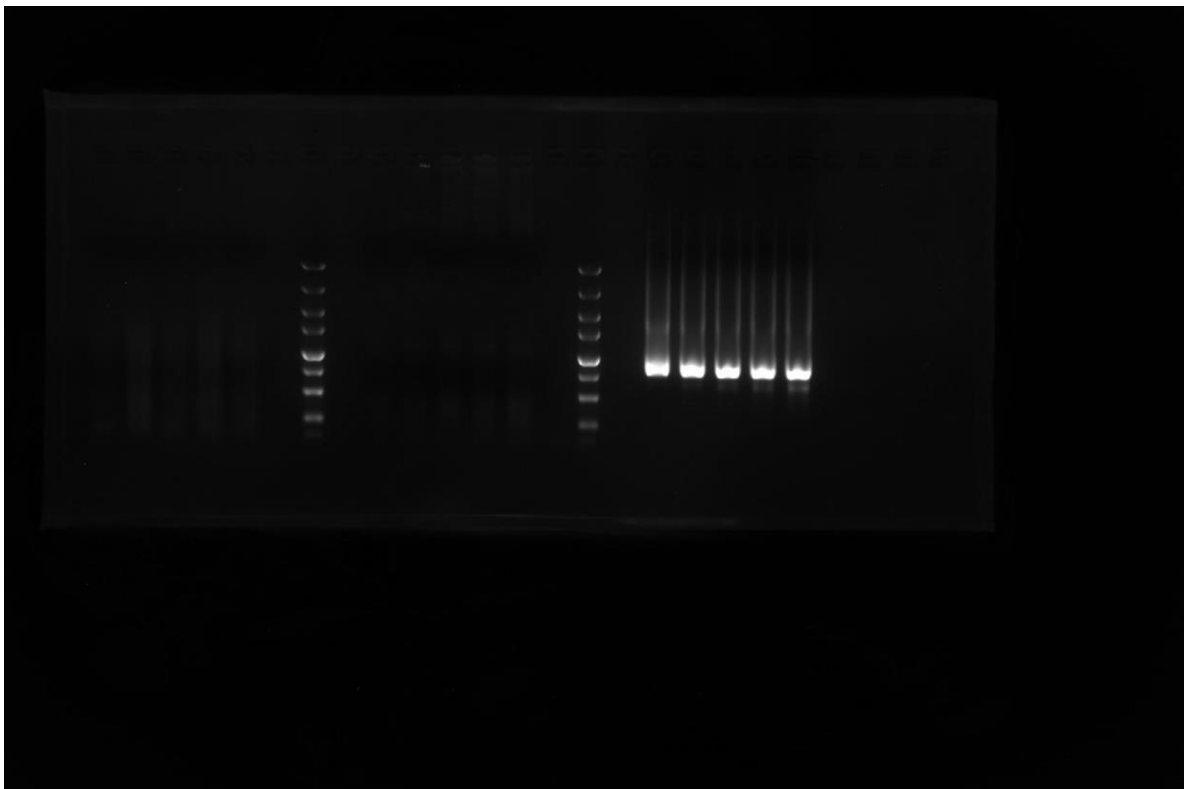

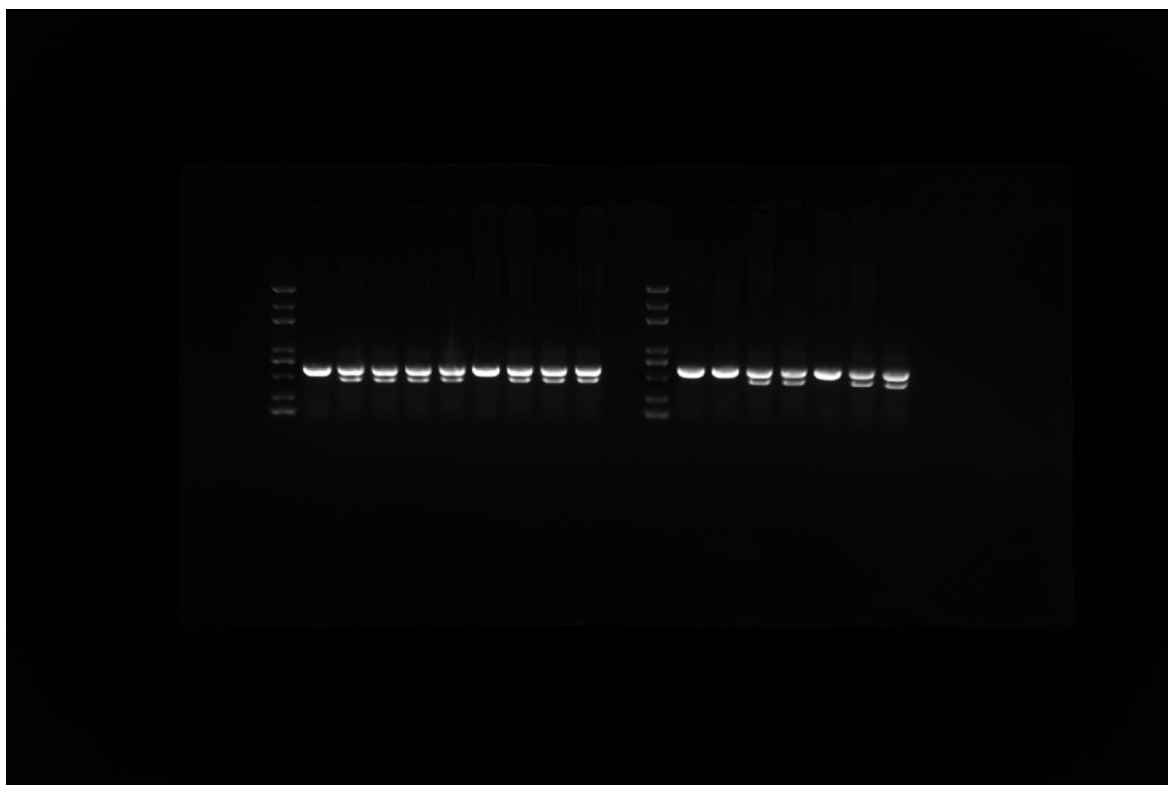

Figure S1: the WB full membrane and the full images of agarose gel electrophoresis of Figure 1

Supplement: Supplementary file 1 [file vetsci-10-00300-s001.zip › Figure S1.pdf]
